# Corrosion Product Film-Induced Stress Facilitates Stress Corrosion Cracking

**DOI:** 10.1038/srep10579

**Published:** 2015-06-11

**Authors:** Wenwen Wang, Zhiliang Zhang, Xuechong Ren, Yongjun Guan, Yanjing Su

**Affiliations:** 1 Corrosion and Protection Centre, Key Laboratory for Environmental Fracture (MOE), University of Science and Technology Beijing, Beijing 100083, China; 2 Department of Structural Engineering, Norwegian University of Science and Technology (NTNU), Richard Birkelands vei 1a, N-7491 Trondheim, Norway; 3 Beijing Institute of Aeronautical Materials, Beijing 100095, China

## Abstract

Finite element analyses were conducted to clarify the role of corrosion product films (CPFs) in stress corrosion cracking (SCC). Flat and U-shaped edge-notched specimens were investigated in terms of the CPF-induced stress in the metallic substrate and the stress in the CPF. For a U-shaped edge-notched specimen, the stress field in front of the notch tip is affected by the Young’s modulus of the CPF and the CPF thickness and notch geometry. The CPF-induced tensile stress in the metallic substrate is superimposed on the applied load to increase the crack tip strain and facilitate localized plasticity deformation. In addition, the stress in the CPF surface contributes to the rupture of the CPFs. The results provide physical insights into the role of CPFs in SCC.

Stress corrosion cracking (SCC) is an event that is caused by the combined effect of a stress and corrosive environment. Corrosion product films (CPFs), such as a passive film, an oxide film or a de-alloyed layer, usually form on the surfaces of a specimen and/or at the crack tip during SCC. A residual stress will be generated in the CPFs during their growth. Consequently, a stress (referred to as the CPF-induced stress) will be induced in the metallic substrate to balance the residual stresses in the CPFs^1^. Experimental and theoretical studies have demonstrated that the CPF-induced stress is usually tensile and the maximum tensile stress exists at the interface between the CPF and the metallic substrate^1^,^2^,^3^ on the matrix side. Systematic studies^4^,^5^,^6^,^7^,^8^,^9^,^10^,^11^,^12^,^13^,^14^,^15^,^16^ have revealed that the dependence of the CPF-induced stress on the anion concentration^4^, the pH value^5^ of the solution, and the applied potential^6^ and hydrogen concentration in the specimens^7^ corresponds with the SCC susceptibility results from slow strain rate tests. For various experimental conditions, a larger CPF-induced stress produces higher SCC susceptibility. When the CPF-induced stress changes from tensile to zero or compressive in certain circumstances, the SCC ceases^6^. The same behaviour has been observed in many SCC systems, such as *α*-Ti in methanol^6^, stainless steel in boiling MgCl_2_^8^, brass and copper in an ammoniacal solution^9^ and low alloy steel in marine environments^10^. Based on this experimental evidence, many researchers have concluded that the CPF-induced stress serves a critical role in SCC^11^.

SCC susceptibility is controlled by the initiation and propagation of stress corrosion cracks. The formation and rupture of CPFs repeatedly occurred during the propagation of the stress corrosion cracks^17^. Based on the CPF-rupture-induced SCC mechanism^18^, the growth rate of the CPF, the critical thickness of the CPF to rupture, the CPF-induced stress, and the CPF rupture frequency are the dominant factors that affect the susceptibility to SCC. The majority of SCC models have been devoted to the mechanical processes induced by the applied load at the crack tip^19^,^20^. The effect of CPFs on the stress field and the plastic deformation in the crack tip vicinity are not well understood, as the physical and analytical investigation of the crack tip with dissimilar material layers is challenging. However, this understanding can be improved using the finite element method (FEM), which has become an accepted analysis technique in many areas of engineering and enables stress and strain analyses to be accurately performed compared with existing classical theoretical methods. The application of the FEM in corrosion research and crack simulation has gained popularity^21^,^22^,^23^,^24^,^25^,^26^,^27^,^28^.

Regarding pitting, Turnbull *et al.*^21^ conducted a finite element analysis to evaluate the stress and strain distribution associated with a single corrosion pit in a cylindrical steel specimen that was remotely stressed in tension. Pidaparti *et al.*^22^,^23^ employed FEM to simulate the morphology and predict the stress buildup created by pits; the results indicated that the stress is responsible for possible crack nucleation. For crack initiation in a corrosion field, Wenman *et al.*^25^ employed the FEM to correlate real transgranular stress corrosion cracks grown in a boiling MgCl_2_ environment and offered explanations for observed morphologies to support proposed crack growth models of this system. Using the FEM, Kamaya *et al.*^26^ deduced that crack initiation is influenced not only by bulk stress applied at the end of the body but also by local stress formed around pre-existing cracks. Vankeerberghen *et al.*^27^,^28^ modelled the crack propagation rate of type 316 stainless steel in boric acid-lithium hydroxide solutions in pressurized-water nuclear reactors relevant conditions.

The formation of a CPF involves either the inward diffusion of anions or the outward immigration of cations. If the cations move outward at a faster rate than the anions move inward, a CPF grows at the film/solution interface with numerous cation vacancies remaining on the metal side, which will produce a tensile stress at the inner interface and a compressive stress in the CPF^17^. Copper and its alloys in an ammoniacal solution are typical SCC systems that were extensively applied to explore the mechanism of SCC. A CPF will form on the copper or copper alloy surface during SCC. Our previous experiments and FEM analyses revealed that compressive stress and tensile stress can be generated in the CPF and the copper substrate, respectively, due to the volume difference between the ionized metal and the oxide. The experimental results have demonstrated that a compressive stress with a magnitude of approximately 1 to 1.5 GPa can be generated in the tarnish film of copper in an ammoniacal solution^1^. In this study, the SCC of copper in an ammoniacal solution was considered to be a finite element model, and the distributions of the CPF-induced stress in the flat and U-shaped edge-notched specimens were simulated. The effect of the properties of the CPFs and the geometric size of the notch on the CPF-induced stress field around a notch tip covered by a CPF are systematically examined. The physical insights of a CPF-induced stress on a CPF rupture, the stress corrosion crack tip strain and/or the strain rate of stress corrosion cracks and the propagation rate of stress corrosion cracks are discussed.

## Results and Discussion

**Figure 1:**
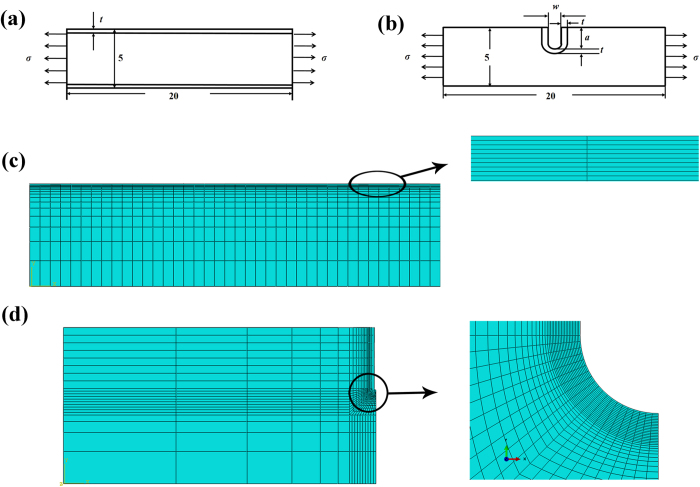
Geometries, dimensions and FEM meshes of the flat and U-shaped edge-notched specimens. (**a**) Dimensions of the full-sized flat model with thickness *B* = 0.4 (unit: mm). (**b**) U-shaped edge-notched model with thickness *B* = 0.4 (unit: mm) (**c**) Total mesh of the 1/8 flat specimen and the local mesh in the CPF. (**d**) The total mesh of the 1/4 U-shaped edge-notched model and the detailed notch tip mesh.

In this study, 3D flat and U-shaped edge-notched specimens were analysed. A 1/8 flat model and a 1/4 U-shaped edge-notched model were employed due to their symmetry. The geometries, dimensions and FEM meshes of the flat and U-shaped edge-notched specimens are shown in Fig. 1.

### Stress distributions in CPF and copper substrate of the flat specimen

**Figure 2:**
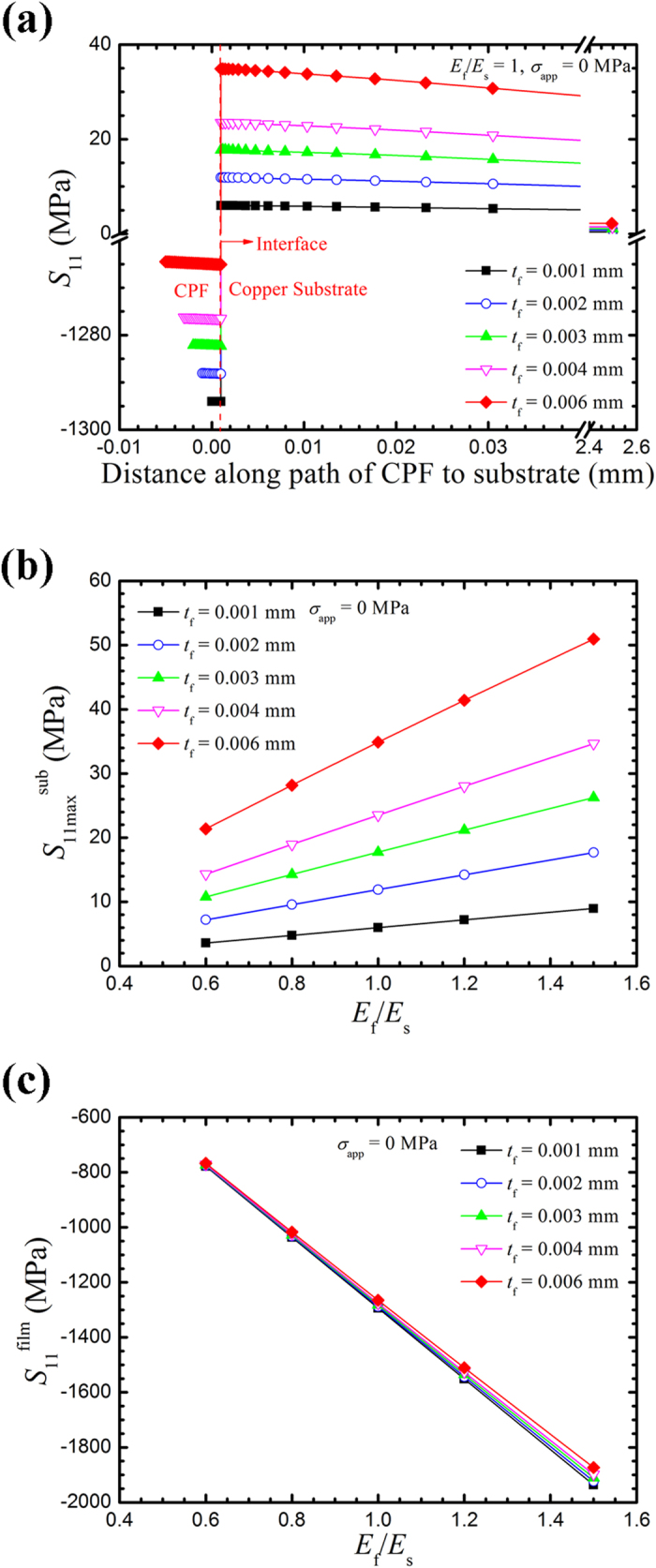
S_**11**_ (the axial stress of the mode I loading direction) in the CPF and copper substrate of the flat specimen. (**a**) *S*_11_ distributions in the middle plane from the CPF to the copper substrate for various CPF thicknesses without applied load. The dashed line represents the interface between the CPF and the copper substrate. (**b**) The variations in the maximum CPF-induced stress in the substrate (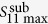) with different CPF thicknesses and Young’s modulus ratios without applied load. (**c**) Dependence of stress in the CPF (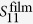) on the CPF thicknesses and Young’s modulus ratios of the CPF to the substrate without applied load.

Figure 2(a) shows the *S*_11_ (the axial stress of the mode I loading direction) distribution along the path in the middle plane from the CPF to the substrate with various CPF thicknesses for the ratio between the CPF Young’s modulus (*E*_f_) and the copper substrate Young’s modulus (*E*_s_), that is, *E*_f_/*E*_s_ = 1 and the applied load σ_app_ = 0 MPa. *S*_11_ is observed to be discontinuous across the interface, a compressive stress uniformly distributes in the CPF, and the absolute value decreases with an increase in CPF thickness. A tensile CPF-induced stress is observed at the interface on the substrate side, which also increases with an increase in CPF thickness. The maximum *S*_11_ appeared at the interface. An eigenstrain analysis^3^ and molecular dynamics simulation^2^ on the stress distribution in the de-alloyed layer and substrate region revealed that the maximum tensile stress existed at the interface, which is consistent with our FEM results.

The maximum values of CPF-induced stress in the substrate (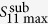) and the stress in the CPF (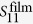), as a function of the Young’s modulus ratio between the CPF and the substrate *E*_f_/*E*_s_ for different CPF thicknesses are shown in Fig. 2(b). Figure 2(b) quantitatively summarizes the dependence of the maximum CPF-induced stress 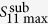 in the copper substrate on the Young’s modulus ratio for various CPF thicknesses. The results conclude that 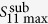 increases with an increase in the CPF Young’s modulus. Δ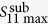 increases with an increase in CPF thickness. These findings indicate that a harder CPF produces a high stress in the substrate, regardless of the CPF thickness. As shown in Fig. 2(c), the stress in the CPF is compressive and the absolute value of 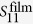 increases with the CPF Young’s modulus and decreases with the CPF thickness. Δ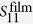 between two different CPF thicknesses increases with an increase in the CPF Young’s modulus.

### The stress distribution in front of the U-shaped notch tip

**Figure 3:**
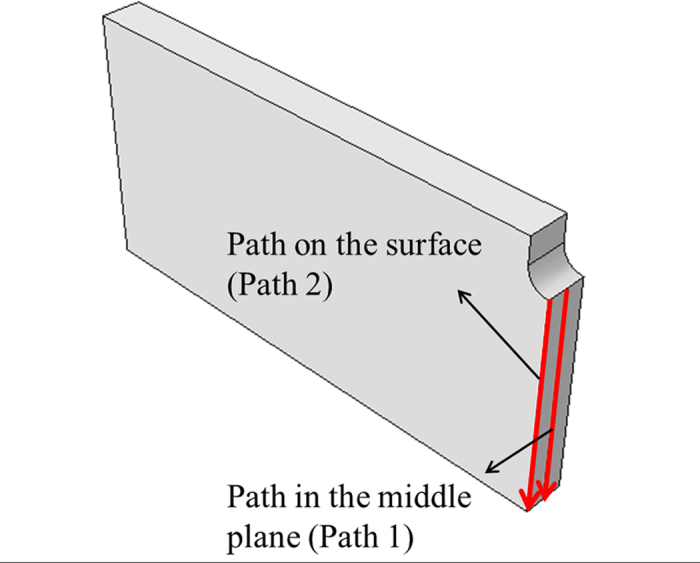


**Figure 4:**
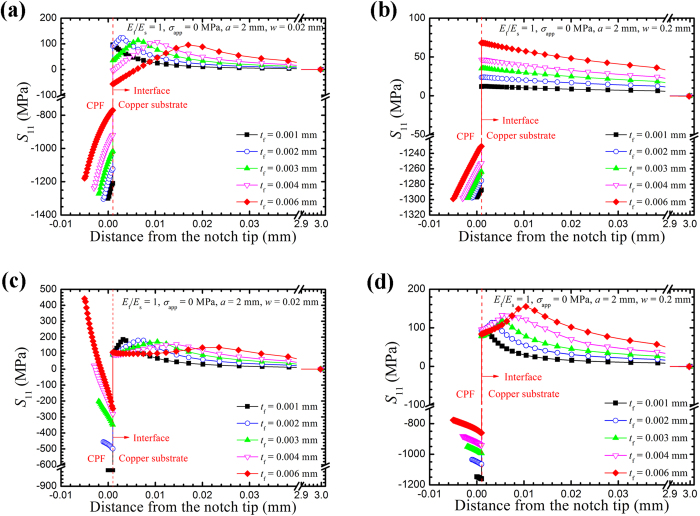
S**_11_** distributions with different CPF thicknesses from the notch tip to the copper substrate in a 1/4 U-shaped edge-notched specimen. (**a**) A small notch opening of 0.02 mm along path 1. (**b**) A wide notch opening of 0.2 mm along path 1. (**c**) A small notch opening of 0.02 mm along path 2. (**d**) A wide notch opening of 0.2 mm along path 2. The dashed line represents the interface between the CPF and the copper substrate.

**Figure 5:**
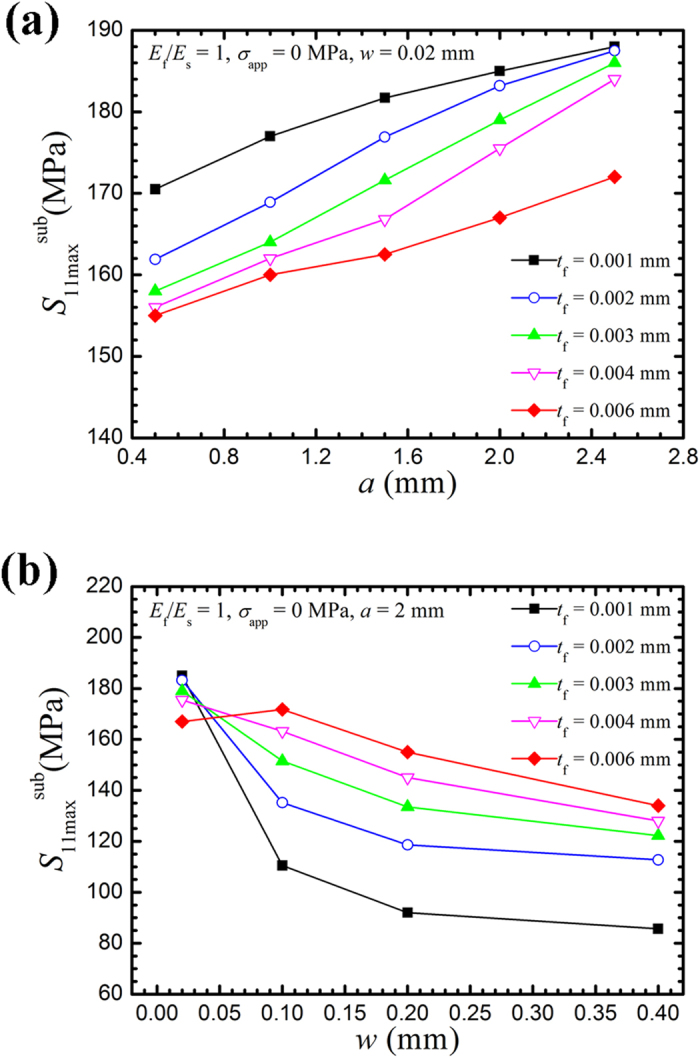
Variation in the maximum CPF-induced stress in the substrate (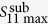) with the notch geometries. (**a**) Variation in 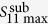 with different notch depths and CPF thicknesses. (**b**) Variation in 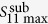 with different notch openings and CPF thicknesses.

**Figure 6:**
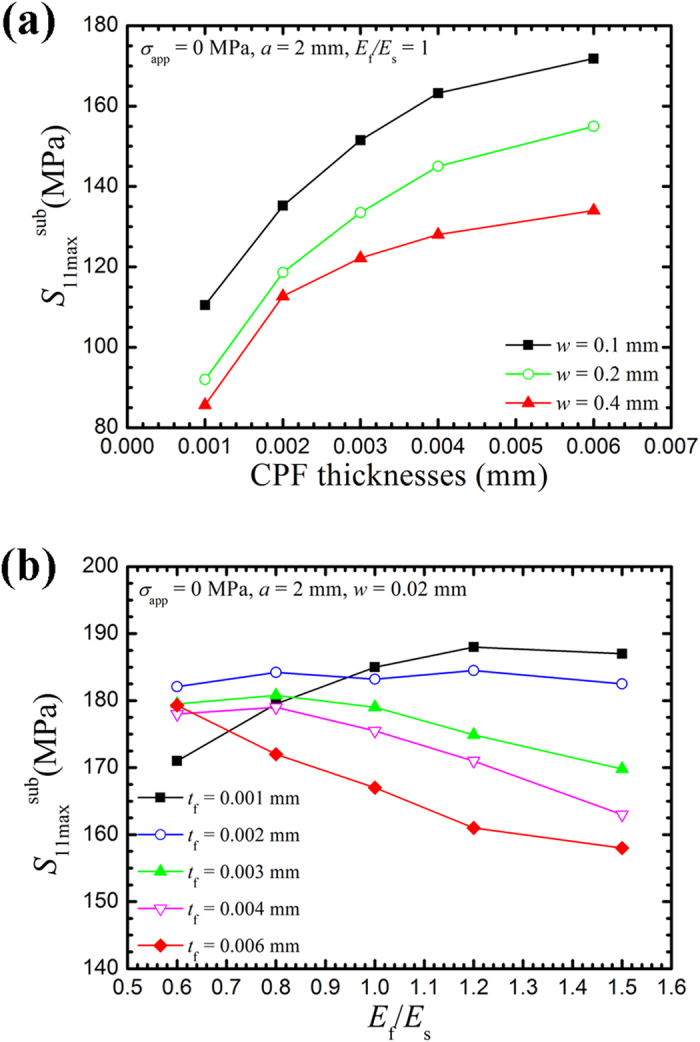
Variation of the maximum CPF-induced stress in the substrate (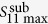) with different CPF parameters. (**a**) Variations in 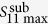 with different CPF thicknesses for an applied load of σ_app_ = 0 MPa. (**b**) Variations of 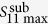 with different values of the Young’s modulus ratio *E*_f_/*E*_s_ without applied load.

**Figure 7:**
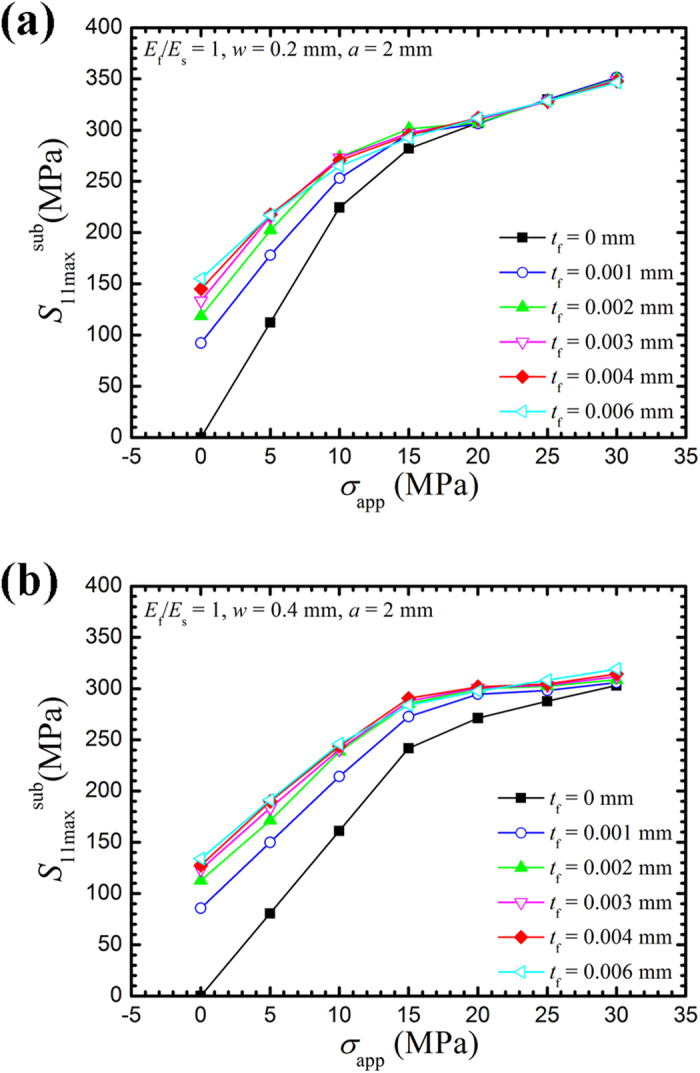
Correlation of the maximum CPF-induced stress in the substrate (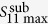) with various applied loads. (**a**) Variations in 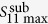 with different CPF thicknesses for a notch opening of 0.2 mm. (**b**) Variations in 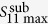 with different CPF thicknesses for a notch opening of 0.4 mm.

The stress at the notch tip governs the initiation and propagation of the stress corrosion cracks. A tensile stress concentration in front of the notch tip is generated by the geometric effect of the notch and the residual stress in the CPF. In the analysis of the results, stress distributions from the CPF surface at the notch tip to the substrate are calculated for the notched sample along the middle plane (referred to as path 1) and the surface (referred to as path 2), as shown in Fig. 3. For the U-shaped edge-notched specimen covered by a CPF, the opening stress (*S*_11_) distributions for various values of *t*_f_ (CPF thickness), *E*_f_, *a* (notch depth), *w* (notch opening) when the CPF expands are shown in Figs. 4, 5, 6, 7.

In the absence of an external load, the *S*_11_ distribution from the CPF at the notch tip to the substrate along path 1 of a narrow notch opening of 0.02 mm is shown in Fig. 4(a). Similar to Fig. 2(a), *S*_11_ is discontinuous and a tensile CPF-induced stress occurs in the substrate. However, the maximum CPF-induced stress along path 1 in the substrate appears near the interface of the CPF/substrate. This stress initially increases and then slightly decreases with an increase in CPF thickness. The location of the maximum CPF-induced stress along path 1 moves away from the interface when the CPF thickness increases. In addition, this stress is several times higher than the stress in flat specimens with the same CPF parameter. In the middle of the specimen, the stress in the CPF is compressive and the absolute value decreases with an increase in CPF thickness and decreasing distance to the interface.

*S*_11_ distributions in the middle plane with a larger notch opening of 0.2 mm are shown in Fig. 4(b). Unlike the *S*_11_ distributions of a small notch opening of 0.02 mm, the maximum CPF-induced stress in this case appeared at the interface and increases with an increase in CPF thickness. The stress in the substrate is lower than the stress for a small notch opening of *w* = 0.02 mm. Figure 4(c) show the *S*_11_ distributions along path 2 for a notch opening of 0.02 mm and a notch opening of 0.2 mm, respectively. Compared with Fig. 4(a), however, the stress in the substrate is larger than the stress in the middle plane and decreases with an increase in CPF thickness. The stress in the CPF changes from compressive to tensile with an increase in CPF thickness. Conversely, no tensile stress is observed in the CPF in Fig. 4(d) and the absolute value decreases with an increase in CPF thickness. The maximum CPF-induced stress along path 2 increases with an increase in CPF thickness.

We can conclude that the CPF-induced stress increases with an increase in CPF thickness for a notch opening of 0.2 mm but deceases with an increase in CPF thickness for a notch opening of 0.02 mm. In addition, the stress decreases with an increase in notch opening. The comparison of Fig. 4(a) with 4(c),(d) reveals that the maximum CPF-induced stress along path 2 is higher than the maximum CPF-induced stress along path 1, that is, the CPFs will induce higher stress in the substrate surface than in the inner region of the specimen.

Figure 5 depicts the effect of notch size on the maximum CPF-induced stress in the entire substrate (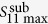, which primarily appeared near the surface). As shown in Fig. 5(a), 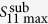 increases with decreasing CPF thickness when the notch depth remains constant. For a certain CPF thickness, 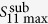
increases with an increase in notch depth. With a thicker CPF (0.006 mm), the maximum CPF-induced stress increases less significantly with the notch depth than the case of a thinner CPF. Figure 5(b) depicts the variation in 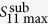 with notch opening. When the CPF thickness is constant, the maximum CPF-induced stress decreases with an increase in the notch opening. For a narrow notch opening of 0.02 mm, 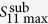 decreases with an increase in CPF thickness. However, for a wide notch equal or larger than 0.2 mm, 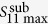 increases with an increase in CPF thickness.

The variation in 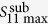 for various CPF thicknesses is shown in Fig. 6(a). With a notch opening equal to or larger than 0.1 mm, 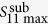 increases with an increase in CPF thickness. In addition, the increase of 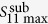 decreases with an increase in CPF thickness, which indicates that a CPF-induced stress will reach a plateau with an increase in CPF thickness. The effect of the Young’s modulus on 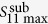 is shown in Fig. 6(b). When *t*_f_ = 0.001 mm, the maximum CPF-induced stress increases with an increase in *E*_f_/*E*_s_; when *t*_f_ = 0.002 mm, 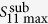 is nearly constant. The maximum CPF-induced stress appears to be insensitive to the CPF Young’s modulus at *t*_f_ = 0.002 mm. With an increase in the CPF thickness, the maximum CPF-induced stress becomes a strong function of the Young’s modulus of the CPF and decreases with an increase in the CPF Young’s modulus. Zhang *et al.*^29^ demonstrated that a critical film thickness exists for a given crack length. When the film is thinner than the critical thickness, a harder film facilitates dislocation emission (which is related to the CPF-induced stress), whereas a softer film hinders the emission. If the film is thicker than the critical thickness, a harder film hinders the emission; a softer film facilitates dislocation emission.

Figure 7 shows the variation of 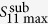 with the applied load for a notch with *a* = 2 mm and *w* = 0.2 and 0.4 mm. 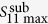 increases with the applied load *σ*_app_; the increase in 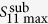 is significantly larger than the increase in the applied loads. This finding indicates that 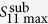 can be significantly magnified with an increase in applied load. For the notches with openings of 0.2 and 0.4 mm, 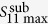 increases with an increase in CPF thickness for the same applied load. We can conclude that the model can achieve the yield stress and increase the opening stress and SCC with a CPF. When the notch opening increases, the effect of the CPF on the CPF-induced stress decreases.

### The CPF rupture process at the notch tip

A CPF will form when the metallic substrate is exposed to environments that cause SCC. SCC can generally be arrested by the formation of a CPF at the crack tip^30^. The rupture of a CPF and its maintenance in the ruptured condition, such that the cracking can proceed, is a prerequisite condition for SCC.

As shown in Fig. 4(a), 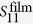 gradually changes from compressive to tensile from the inner region to the surface when the CPF thickness is sufficiently thick and covers the notch tip with a relatively small opening, whereas 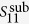 (CPF-induced stress in the substrate) remains consistently tensile. The stress field analysis indicates two possible mechanisms of CPF rupture during SCC. The first mechanism involves a tensile stress that is produced on the surface of the CPF when the CPF grows sufficiently thick at the notch tip with a small opening. In this case, the cracks in the CPF initiate in the CPF on the surface in the specimen, and the stress along the cracks subsequently becomes more concentrated, which would assist the rupture of the CPF. In the second mechanism, localized plastic flow (dislocation emission and motion) of the metal substrate at the crack tip occurs, which is induced by the CPF-induced tensile stress; this mechanism is the mechanism of CPF rupture. The CPF-induced tensile stress that is superimposed on the applied stress to enhance dislocation emission and motion from the crack tip followed by initiation and propagation of SCC has been demonstrated by a series of TEM observations^31^,^32^.

Dislocation emission and motion from the crack tip is related to the resolved shear stress. According to Schmid’s law, the resolved shear stress is proportional to the normal stress at the crack tip, which is the sum of the applied model I stress and the CPF-induced stress in this study. This relationship indicates that the presence of the CPF-induced stress will increase the resolved shear stress due to the growth of a CPF at the crack tip. After attaining the critical resolved shear stress, localized plastic deformation of the metallic substrate can initiate CPF rupture. This process explains why the critical applied load, which induces localized plastic deformation and causes crack initiation during SCC, is significantly lower than the critical applied load in air.

**Figure 8:**
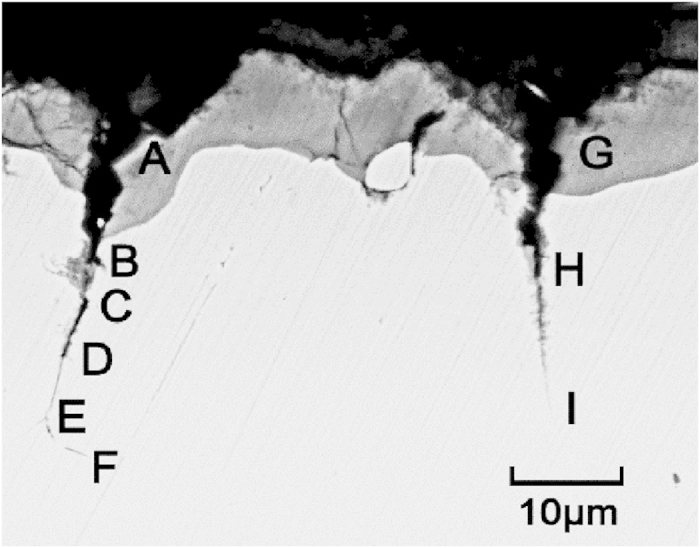
Initiation and propagation of the stress corrosion cracks in the stress-free brass specimen, which were induced by the rupture of the CPFs. A-F and G-H represent two cracks; points A and G are located on the CPF and points B-F and G are located on the substrate.

The CPF-induced stress ruptures the CPF and then induces the propagation of the stress corrosion cracks; this process was experimentally demonstrated. When a stress-free flat brass specimen, which was fully annealed at 320 °C for two hours followed by electrochemical polishing to remove the residual stress of the surface, was immersed in Mattsson’s solution for 120 h, cracks appeared on the surface of the specimen, as shown in Fig. 8, where A-F and G-H represent two cracks. The crack initiates in the CPF and then propagates to the substrate; points A and G are located on the CPF and points B-F and G are located on the substrate. The CPF-induced stress in the brass specimen increases as the CPF grows. After the critical value is attained, localized plastic flow will occur in a metallic substrate to rupture the CPF. The CPF is periodically ruptured by the localized plastic flow of the metallic substrate induced by the CPF-induced stress to facilitate the initiation and propagation of stress corrosion cracks^9^.

### The CPF-induced stress facilitates SCC

SCC is caused by localized oxidation that is enhanced by stress/strain at the crack tips. Based on the slip/dissolution-oxidation model, the rupture of the CPF at the crack tip dominates the crack growth rate (*da*/*dt*) as described in the following formula^33^


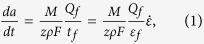


where *M* and *ρ* are the atomic weight and density of the corroding metal; *z* is the change in charge due to oxidation; *F* is Faraday’s constant; *t*_f_ is the time between CPF rupture events in the CPF at the crack tip, which is related to the strain between rupture events (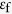) and the local crack tip strain rate (CTSR, 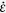) as *t*_f_ = 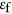/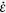; and *Q*_f_ is the integral of the current density-time curve between fracture events. Eq. (1) yields the average crack propagation rate in one CPF rupture event. However, it has been employed to calculate crack growth rates during some stress corrosion processes.

The argument is focused on the local CTSR, which is generally considered to be dominated by the applied loading condition and the crack geometries. However, by considering the CPF-induced stress at the crack tip for the condition of small-scale yielding, the stress intensity factor of a stress corrosion crack can be written as


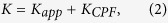


where *K*_app_ and *K*_CPF_ are the stress intensity factor of the applied load and the CPF-induced stress, respectively.

Rice *et al.*^34^ proposed a crack tip strain rate model due to a steady increase in the applied stress intensity factor, as written in Eq. (3)


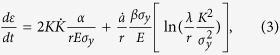


where *α*, *β*, *λ* are constants in the plastic strain calculation and *r* is the distance from a notch/crack tip, 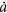 = *da*/*dt*, 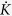 = *dK*/*dt*.

A slowly growing stress corrosion crack under a constant applied load/displacement can be considered to be a quasi-static crack. In this circumstance, the applied stress intensity factor between CPF rupture events can be approximated to be constant, that is, 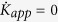. By substituting Eq. (2) into Eq. (3),


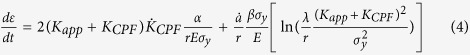


The results from Eqs. (1) and (4) conclude that the CPF-induced stress will promote the stress corrosion crack growth rate by superimposing on the applied stress to increase the CTSR.

As shown in Eq. (4), both *K*_CPF_ and 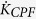, which are proportional to the CPF-induced stress and the increase rate of the CPF-induced stress (i.e., the growth rate of the CPF), respectively, dominate CTSR. A higher value of CTSR indicates a higher CPF rupture frequency (small *t*_f_ in Eq. (1)), which consequently induces a higher stress corrosion crack growth rate. As shown in Fig. 6(a), 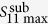 increases with an increase in CPF thickness and the increase rate of the CPF-induced stress is positive at the beginning of the formation of the CPFs at the notch tip, which implies that the CTSR increases with the growth of the CPF at the beginning of the formation of the CPF. Therefore, the growth of a CPF at the crack tip, which can rapidly induce a higher tensile stress in the substrate, is a prerequisite condition for SCC.

The CPF is usually an oxide, which is brittle, and the crack propagation rate in a CPF is significantly faster than the crack propagation rate in a metallic substrate. The crack propagation rate (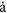) in Eq. (4) should be considered to be the crack propagation rate in a CPF. However, this parameter has always been considered to be the crack propagation rate in the metallic substrate in previous discussions. The crack propagation rate in a CPF during SCC should be the instantaneous crack propagation rate of the stress corrosion crack propagation. A brittle crack is nucleated and rapidly propagates in the CPF. The brittle crack is arrested at the interface between the CPF and the metallic substrate or in the substrate after propagating for some distance in the substrate. For crack arrest to occur, many dislocations must be nucleated at the tip of the moving crack due to the impacting effect of the fast propagation of the brittle crack, which increases the plastic strain in front of the crack.

Finite element analyses have been performed to clarify that the CPF can induce a tensile stress in the metallic substrate when the film expands. The stress field in front of the notch tip is influenced by the CPF Young’s modulus, the CPF thickness, and the notch geometry. The CPF-induced stress can assist the applied load to facilitate localized plastic flow and enhance the strain rate of the crack tip by increasing the resolved shear stress. The tensile stress in the CPF at the surface can contribute small cracks in the CPF. Both stresses facilitate crack initiation in the SCC.

## Methods

The finite element software ABAQUS was employed to analyse the SCC crack tip zone. A 3D finite-element model was employed to investigate the alteration of the crack tip stress field, which was produced from the coverage of the CPF. The flat model with a full-sized test coupon of 20 × 5 × 0.4 mm^3^ was employed. The size of the U-shaped edge-notched model was equivalent to the size of the flat specimen, and a 1/4 model with dimensions of 10 × 5 × 0.2 mm^3^ was employed due to its symmetry. Five CPF thicknesses—*t*_f_ = 0.001, 0.002, 0.003, 0.004, 0.006 mm—were utilized. Considering the symmetry, only a 1/8 model was employed. The 3D model was constructed with eight-node linear brick elements (C3D8R).

Copper was considered for the metallic substrate material in this study, as its plastic properties were obtained from our previous study^35^. The properties of the copper substrate were modelled with Young’s modulus *E*_*s*_ = 130 GPa, Poisson’s ratio *ν* = 0.3 and yield strength *σ*_ys_ = 250 MPa. The load was applied perpendicular to the notch (mode I loading).

To simulate the CPF-induced stress in the FEM, the CPF was assigned to have a nonzero thermal expansion coefficient, whereas a zero value was assigned to the copper substrate. A similar method has been employed to introduce the residual stresses induced by elastic anisotropy^36^,^37^,^38^ and the welding process^39^,^40^, in which the thermal expansion coefficients had no specific physical meaning. From the assumed CPF property, when Δ*T* > 0 (in which Δ*T* corresponds to the temperature change in the simulation process), the CPF expands to simulate the compressive stress in the CPF. A suitable expansion coefficient was selected to simulate the stress in the CPF within a reasonable range at Δ*T* = 1. When the notch and CPF dimensions changed, the mesh density around the notch was slightly altered to ensure consistency in the size of the elements created around the notch, which indicates that the meshes created for the cases with different notch sizes will not be completely identical but should yield similar levels of accuracy.

## Additional Information

**How to cite this article**: Wang, W. *et al.* Corrosion Product Film-Induced Stress Facilitates Stress Corrosion Cracking. *Sci. Rep.* **5**, 10579; doi: 10.1038/srep10579 (2015).
